# A 7-Year Study of the Durability of Improvements in Pain, Physical Function, and Work Productivity After Roux-en-Y Gastric Bypass and Sleeve Gastrectomy

**DOI:** 10.1001/jamanetworkopen.2022.31593

**Published:** 2022-09-14

**Authors:** Wendy C. King, Amanda S. Hinerman, Gretchen E. White

**Affiliations:** 1 Department of Epidemiology, School of Public Health, University of Pittsburgh, Pittsburgh, Pennsylvania; 2 Division of General Internal Medicine, School of Medicine, University of Pittsburgh, Pittsburgh, Pennsylvania

## Abstract

**Importance:**

Bariatric surgical procedures are associated with clinically important improvements (CIIs) in pain and physical function. However, there are declines in initial improvement by the third postoperative year, and the long-term durability of improvements are not well-described.

**Objective:**

To evaluate the durability of improvements in pain and physical function through 7 years after Roux-en-Y gastric bypass (RYGB) or sleeve gastrectomy (SG).

**Design, Setting, and Participants:**

This study is part of the Longitudinal Assessment of Bariatric Surgery–2 (LABS-2), a cohort study at 10 US hospitals. Adults with severe obesity (ie, body mass index of 35 or greater) undergoing bariatric surgery were assessed preoperatively (2006-2009) and followed up annually for as long as 7 years or until 2015. Of 1829 participants who underwent RYGB or SG in LABS-2, 338 were excluded from this study because they had a follow-up period of less than 5 years. Analysis of participants who underwent RYGB or SG and completed research assessments preoperatively and postoperatively for 5 to 7 years was conducted from March to April 2022.

**Main Outcomes and Measures:**

Preoperative-to-postoperative CIIs in pain and physical function scores from the 36-Item Short Form Health Survey and the Western Ontario McMaster Osteoarthritis Index, and 400-meter walk time, using previously established thresholds; and remission of mobility deficit, ie, inability to walk 400 meters in 7 minutes or less.

**Results:**

A total of 1491 individuals were included, with 1194 (80%) women; 59 (4%) Hispanic, 164 (11%) non-Hispanic Black, and 1205 (82%) non-Hispanic White individuals; a preoperative median (IQR) age of 47 (38-55) years; and a preoperative median (IQR) body mass index of 47 (42-52). Between 3 and 7 years after surgery, the percentage of participants with preoperative-to-postoperative CIIs in bodily pain decreased from 50% (95% CI, 48%-53%) to 43% (95% CI, 40%-46%), in physical function from 75% (95% CI, 73%-77%) to 64% (95% CI, 61%-68%), and in 400-meter walk time from 61% (95% CI, 56%-65%) to 50% (95% CI, 45%-55%). Among participants with a preoperative mobility deficit, remission decreased from 50% (95% CI, 42%-57%) to 41% (95% CI, 32%-49%), and among participants with severe knee or hip pain or disability, the percentage with CIIs in knee and hip pain and function decreased (eg, hip pain: from 77% [95% CI, 72%-82%] to 65% [95% CI, 58%-72%]; knee function: from 77% [95% CI, 73%-82%] to 72% [95% CI, 67%-77%]).

**Conclusions and Relevance:**

In this cohort study, despite decreases in preoperative-to-postoperative improvements across follow-up, CIIs in perceived bodily and joint-specific pain and in self-reported and objectively measured physical function ranged from 41% to 72%, depending on the measure and subgroup, 7 years after surgery, suggesting that RYGB and SG are commonly associated with long-term CIIs in pain and physical function.

## Key Points

**Question:**

Do adults experience long-term clinically important improvements (CIIs) in pain and physical function after Roux-en-Y gastric bypass (RYGB) and sleeve gastrectomy (SG)?

**Findings:**

Among 1491 US adults who underwent RYGB or SG, 43% had CIIs in bodily pain and 64% had CIIs in physical function 7 years after surgery. Most participants with preoperative symptoms indicative of osteoarthritis of the knee or hip had CIIs (65%-72%), and 41% of participants with an objectively defined mobility deficit no longer had one.

**Meaning:**

In this study, despite aging, participants commonly experienced CIIs in bodily and joint-specific pain and physical function 7 years after undergoing RYGB or SG.

## Teaser

This cohort study evaluates the durability of improvements in pain and physical function through 7 years after Roux-en-Y gastric bypass (RYGB) or sleeve gastronomy (SG).

## Introduction

Severe obesity is associated with significant joint pain and impaired physical function,^1,2^ which contribute to impaired health-related quality of life (HRQoL) and impaired work productivity.^3,4,5,6,7^ Adults scheduled to undergo bariatric surgery name physical pain, difficulties moving (eg, lifting, bending, walking, climbing stairs) and performing daily tasks (ie, personal hygiene, household chores), and maintaining employment or performing job duties as motivators for surgery, with improvements in mobility, functionality, and employment as goals.^8^

On average, modern-day bariatric surgical procedures result in substantial weight loss and improvements in comorbidities, HRQoL, joint pain, physical function, and work productivity.^9,10,11,12^ Improvements are generally greatest 1 to 2 years after surgery, with some backsliding by the third year.^9,12,13,14,15,16^ While the average net effect of bariatric surgery is still positive, weight, cardiovascular and diabetes parameters, and HRQoL generally worsen 3 to 7 years after surgery.^12,14,17^ Most evaluations of joint pain, physical function, and work productivity following surgery are limited to no more than 2 years follow-up or evaluate outdated procedures^10,11,18^; thus, the durability of improvements following current bariatric surgical procedures is unclear. Evaluating longer-term changes in these important clinical and economic outcomes is needed to inform patient expectations, clinical care, and health insurance companies.

Investigators of the Longitudinal Assessment of Bariatric Surgery–2 (LABS-2), a large multicenter US cohort study, previously reported preoperative-to-postoperative changes in bodily and joint-specific pain, physical function, and work productivity 1 to 3 years after bariatric surgery.^13,15^ This report addresses the durability of improvements, focusing on changes between 3 and 7 years and 7 years’ after surgery vs preoperative status, following Roux-en-Y gastric bypass (RYGB) and sleeve gastrectomy (SG), the 2 most common bariatric procedures today.^9^

## Methods

The LABS-2 study (NCT00465829) recruited adults undergoing first-time bariatric surgical procedures for clinical care at 10 US centers.^19^ Each center obtained institutional review board approval; participants provided written informed consent. A total of 2458 participants completed assessments within 30 days before surgery (2006-2009) and annually for as many as 7 years after surgery, through January 2015. This report is limited to participants who underwent RYGB or SG and completed postoperative assessments for at least 5 years. Of the 1829 participants who underwent RYGB or SG, 338 were excluded from this report because they had a follow-up of less than 5 years, leaving 1491 (82%).

The LABS-2 protocol is available online.^20^ A description of research assessments has been published.^19^ Measures included in this report are described in the eAppendix in the Supplement. Self-reported race and ethnicity were collected as descriptors of the study sample to address generalizability of the study findings. Race was set to missing for participants who did not self-report their race as 1 or more of the following options: American Indian or Alaska Native, Asian, Black or African American, Native Hawaiian or other Pacific Islander, and White.

Primary end points were preoperative-to-postoperative clinically important improvements (CIIs) in bodily pain and physical function, assessed with the Medical Outcomes Study 36-Item Short-Form Health Survey (SF-36) (ie, ≥5 point increase in the norm-based scores^21^) and 400-meter walk time (ie, ≥24-second decrease^22^). Secondary end points were CIIs in knee pain, hip pain, knee function, and hip function, assessed with the Western Ontario McMaster Osteoarthritis Index (WOMAC) (ie, ≥9.7 pain point or ≥9.3 function point decrease in score^23^), and remission of mobility deficit (ie, inability to walk 400 meters in 7 minutes or less^24^). We also evaluated changes in a variety of pain, physical function, and work productivity measures.

### Statistical Analysis

Analyses were conducted using SAS version 9.4 (SAS Institute) in March and April 2022. Potential selection bias was examined by comparing preoperative characteristics of those in the analysis sample with those who were excluded due to missing data using the Pearson χ^2^ test for categorical variables and the Wilcoxon rank-sum test for continuous variables.

Longitudinal analyses were performed using mixed models (binary, ordinal, or linear via maximum likelihood) with a person-level random intercept and time (assessment) as a discrete conditional likelihood, controlling for preoperative factors associated with missing follow-up data (eg, site, age).^13^ Analysis of 400-meter walk time was limited to participants who completed the walk preoperatively, WOMAC scores to participants with symptoms indicative of osteoarthritis (severe or extreme rating on ≥1 item in the relevant joint^25^) preoperatively, mobility deficit remission to participants with a preoperative mobility deficit, and work productivity to participants who worked for pay throughout the study period.^15^ Heart rate was set to missing at time points when participants reported taking β-blockers. Modeled means or percentages and 95% CI are reported by assessment. To assess long-term changes, we made pairwise comparisons between the preoperative and year-7 assessments. To assess stability beyond 3 years after surgery, we limited the data set to years 3 to 7 and tested for linear and quadratic trends with time (days since surgery) as conditional likelihood. All *P* values are 2-sided and reported to guide interpretation of results.^26,27^

Too few participants underwent SG (51 individuals) to stratify analyses by surgical procedure. However, descriptive statistics for the SG subgroup were computed.

## Results

### Preoperative Characteristics

A total of 1491 individuals were included, with 1194 (80%) women; 59 (4%) Hispanic, 164 (11%) non-Hispanic Black, and 1205 (82%) non-Hispanic White individuals; a preoperative median (IQR) age of 47 (38-55) years; and preoperative median (IQR) body mass index (BMI; calculated as weight in kilograms divided by height in meters squared) of 47 (42-52) (eTable 1 in the Supplement). Approximately half (764 of 1490 [51%]) had sleep apnea, 526 of 1464 (36%) diabetes, 386 of 1457 (27%) asthma, 127 of 1460 (9%) cardiovascular disease, 125 of 1490 (8%) venous edema with ulcerations, and 16 of 1489 (1%) history of stroke (eTable 1 in the Supplement). Approximately 30% of participants had a history of back or leg surgery (275 of 1368 [20%] knee; 111 of 1367 [8%] back; 76 of 1366 [6%] ankle; and 47 of 1365 [3%] hip). Overall, 557 of 1310 (43%) had a mobility deficit, 459 of 1088 (42%) symptoms indicative of 73osteoarthritis of the knee, and 347 of 1089 (32%) symptoms indicative of osteoarthritis of the hip (eTable 1 in the Supplement). More than two-thirds (953 of 1377 [69%]) worked for pay; among nonretired participants, 953 of 1305 (69%) worked for pay. Most participants (1440 [97%]) underwent RYGB.

Compared with participants included in this report, the 338 excluded due to missing data were similar with respect to sex, race and ethnicity, household income, BMI, comorbidity burden, self-reported and objectively measured walking capacity, and SF-36 bodily pain and physical function scores (eTable 1 in the Supplement). However, excluded participants were younger, more likely to smoke in the past year, and less likely to report symptoms indicative of osteoarthritis of the knee.

### Age, Weight, Joint Operations, and Employment Across Follow-up

Age, weight loss, BMI, back and leg operations, and work status by follow-up assessment are reported in eTable 2 in the Supplement. At 7 years, the median (IQR) age was 52 (45-62) years, median (IQR) weight loss was 28% (20%-36%) of preoperative weight, and median (IQR) BMI was 34 (29-39). Among 1112 participants reporting whether they had a past-year surgery at 4 or more postoperative assessments, more than one-fifth (252 [23%]) underwent back or leg surgery during follow-up (160 [14%] knee, 97 [9%] back, 51 [5%] ankle, and 69 [6%] hip). At 7 years, 514 of 838 (61%) worked for pay; among nonretired participants, 514 of 729 (71%) worked for pay.

### Pain and Physical Function

Tables 1 and 2 show the modeled pain and physical function measures, respectively, by assessment; descriptive statistics are provided in eTable 3 and eTable 4 in the Supplement. Most measures worsened 3 to 7 years after surgery but were better at 7 years than in the preoperative period by varying degrees. For example, the mean SF-36 bodily pain score decreased from 3 to 7 years from 45 (95% CI, 44-45) to 42 (95% CI, 42-43), 3 points higher (ie, better) than the preoperative score (39 [95% CI, 39-40]). In contrast, the SF-36 physical function score decreased from 3 to 7 years from 48 (95% CI, 48-49) to 46 (95% CI, 45-47), 10 points higher than the preoperative score (36 [95% CI, 35-36]). A few measures (prevalence of taking pain medication for back pain, not being able to go to work or school due to back or leg pain, severe walking limitation, mobility aid use, and 400-meter walk completion), which worsened 3 to 7 years after surgery, did not appear to differ at 7 years after surgery vs before surgery.

**Table 1.** Pain Before and After Roux-en-Y Gastric Bypass and Sleeve Gastrectomy Among 1491 Participants^a^

Outcome	Participants, No.	Model-based estimates, % (95% CI)^b^							*P* value	
		Preoperative	Year 1	Year 2	Year 3	Year 4	Year 5	Year 7	Preoperative vs year 7	Years 3 to 7^c^
SF-36 Score, mean^d^										
Bodily pain	1378	39.2 (38.6-39.7)	47.2 (46.6-47.8)	46.1 (45.5-46.7)	44.6 (44.0-45.2)	44.0 (43.4-44.6)	43.4 (42.7-44.0)	42.3 (41.6-43.0)	<.001	<.001
Back or leg pain										
Medication for back pain, past week	1359	35.7 (33.2-38.1)	29.2 (26.9-31.8)	29.1 (26.9-31.6)	32.0 (29.6-35.0)	34.9 (32.2-37.6)	36.2 (33.7-38.7)	37.2 (34.3-39.9)	.40	.008
Medication for leg pain, past week	1358	42.1 (39.5-44.6)	26.6 (24.2-29.0)	28.2 (25.9-30.9)	31.1 (28.6-33.9)	34.0 (31.4-36.8)	34.9 (32.3-37.6)	38.4 (35.0-41.5)	.04	<.001
Back or leg pain interfered with work (outside the home or house work), past 4 weeks										
Not at all	1369	46.3 (44.2-48.6)	70.3 (66.7-74.3)	72.3 (68.3-76.5)	70.5 (66.5-74.6)	69.4 (65.3-73.5)	66.6 (62.8-70.2)	63.9 (59.9-68.7)	<.001	<.001
A little bit		14.5 (13.8-14.8)	10.2 (9.2-10.8)	7.1 (6.3-7.9)	7.9 (7.0-8.8)	8.8 (7.9-9.7)	8.7 (8.0-9.6)	10.6 (9.4-11.3)		
Moderately		14.5 (14.1-14.9)	7.9 (7.0-8.7)	6.9 (5.8-7.8)	8.8 (7.9-9.8)	7.3 (6.4-8.3)	8.5 (7.7-9.3)	9.4 (8.2-10.3)		
Quite a bit		15.2 (14.9-15.7)	7.0 (6.0-7.9)	8.1 (7.0-9.1)	6.5 (5.5-7.3)	9.7 (8.4-10.7)	10.5 (9.5-11.5)	10.2 (8.9-11.3)		
Extremely		9.6 (8.5-10.5)	4.7 (3.5-6.0)	5.6 (4.3-6.8)	6.4 (5.0-7.7)	4.9 (3.9-6.0)	5.7 (4.5-6.8)	6.0 (4.7-7.3)		
Could not go to work or school due to back or leg pain, past 4 weeks	1369	7.3 (6.0-8.7)	3.9 (2.9-5.0)	3.6 (2.6-4.6)	3.6 (2.6-4.8)	4.3 (2.9-5.4)	5.1 (3.9-6.5)	6.4 (4.7-7.7)	.40	.003
Feelings regarding back or leg pain										
Very dissatisfied	1367	26.2 (27.1-26.3)	7.7 (6.7-8.7)	7.7 (7.3-8.1)	8.8 (8.1-9.2)	10.2 (9.3-10.5)	10.7 (9.5-11.3)	12.4 (12.8-13.0)	<.001	.005
Dissatisfied		15.6 (15.4-15.7)	7.9 (6.7-8.2)	7.4 (6.5-7.9)	7.1 (5.8-7.8)	7.4 (6.7-8.3)	8.7 (8.5-9.4)	7.0 (5.9-8.3)		
Somewhat dissatisfied		9.0 (8.6-9.7)	7.5 (6.7-8.0)	7.4 (6.6-8.2)	8.4 (7.9-9.3)	8.3 (7.3-9.5)	9.5 (8.7-10.0)	9.7 (9.1-10.4)		
Neither satisfied or dissatisfied		3.0 (2.2-3.4)	4.3 (3.7-4.8)	4.2 (3.4-5.2)	4.0 (3.4-5.0)	4.5 (4.0-4.9)	4.6 (3.9-5.6)	5.2 (4.8-6.6)		
Somewhat satisfied		1.0 (0.7-1.6)	2.7 (2.4-3.4)	1.8 (1.2-2.4)	2.6 (1.8-3.7)	1.4 (0.6-2.0)	1.5 (0.8-2.4)	3.0 (2.2-3.7)		
Satisfied		1.0 (0.7-1.3)	3.4 (2.7-4.7)	3.0 (2.1-4.3)	2.2 (1.6-2.9)	2.7 (2.0-3.1)	2.2 (1.6-2.7)	2.3 (1.6-3.1)		
Very satisfied		44.2 (45.2-41.9)	66.4 (71.0-62.2)	68.5 (72.9-63.9)	66.9 (71.2-62.2)	65.5 (70.1-61.6)	62.8 (66.9-58.6)	60.3 (63.6-55.1)		
Back pain during 400-m walk	1033	19.2 (16.8-21.4)	7.2 (5.7-8.5)	6.6 (4.9-8.5)	8.8 (6.9-10.8)	10.6 (8.7-12.6)	7.9 (6.2-10.3)	10.8 (8.2-13.3)	<.001	.92
Leg pain during 400-m walk	1033	44.4 (41.5-47.3)	20.0 (17.6-22.4)	19.7 (17.1-22.8)	22.7 (20.0-25.9)	24.6 (21.4-27.4)	25.1 (22.1-28.3)	31.2 (27.3-34.8)	<.001	.005
WOMAC scores, mean^e^										
Knee pain^f^	455	45.6 (43.9-47.1)	22.4 (20.8-24.1)	22.6 (21.0-24.2)	23.5 (21.9-25.2)	23.7 (22.0-25.4)	26.1 (24.1-27.8)	27.3 (25.0-29.5)	<.001	.003
Hip pain	347	45.6 (43.9-47.6)	22.9 (20.9-24.9)	22.8 (20.9-24.8)	23.2 (21.5-25.3)	24.6 (22.6-26.6)	27.5 (25.2-29.4)	28.6 (26.0-31.0)	<.001	<.001

Abbreviations: SF-36, Medical Outcomes Study Short-Form 36 Health Survey; WOMAC, Western Ontario and McMaster Universities Osteoarthritis.

^a^ Back and leg pain during the 400-meter walk were evaluated among those who started the 400-meter walk; knee pain and hip pain were evaluated among those with symptoms indicative of osteoarthritis in the respective joint.

^b^ Adjusted for site and age; eTable 3 in the Supplement presents the observed data.

^c^ *P* values for linear trends are reported given that all *P* values for quadratic trends were .05 or greater.

^d^ Norm-based methods were used to transform scores (mean [SD], 50 [10]) in the general US population. Higher scores indicate less pain.

^e^ Lower scores indicate less pain and better function on a 0 to 100 point scale.

^f^ Excludes 4 of 459 participants with preoperative symptoms of osteoarthritis in the knee due to missing preoperative knee pain score.

**Table 2.** Physical Function Before and After Roux-en-Y Gastric Bypass and Sleeve Gastrectomy Among 1491 Participants^a^

Outcome	Participants,No.	Model-based estimates, % (95% CI)^b^							*P* value	
		Preoperative	Year 1	Year 2	Year 3	Year 4	Year 5	Year 7	Preoperative vs year 7	Years 3 to 7^c^
SF-36 Scores, mean^d^										
Physical function	1382	35.6 (35.0-36.2)	49.6 (49.0-50.1)	49.2 (48.6-49.8)	48.1 (47.5-48.7)	47.3 (46.7-47.9)	46.4 (45.7-47.0)	45.1 (44.4-45.8)	<.001	<.001
Self-reported walking										
Severe walking limitation	1346	7.1 (5.7-8.3)	4.7 (3.5-5.8)	4.3 (3.4-5.5)	5.4 (4.2-6.7)	6.2 (4.9-7.5)	7.6 (6.0-9.2)	7.6 (5.9-9.2)	.60	.02
Mobility aid use	1348	16.1 (14.1-18.2)	9.8 (8.3-11.5)	10.4 (8.9-12.0)	12.2 (10.5-14.2)	12.4 (10.4-14.4)	13.9 (12.0. 16.2)	18.2 (15.4-21.0)	.13	.04^e^
Health limits ability to										
Walk 1 block	1373	41.8 (39.4-44.5)	9.5 (8.0-11.1)	11.1 (9.5-12.8)	11.9 (10.2-14.0)	14.7 (12.8-16.7)	17.1 (15.1-19.2)	20.4 (17.7-22.8)	<.001	<.001
Walk several blocks	1379	67.8 (65.2-70.3)	17.4 (15.5-19.7)	18.5 (16.4-20.7)	22.3 (20.0-24.7)	23.9 (21.7-26.5)	28.1 (25.7-30.5)	31.6 (28.6-34.5)	<.001	<.001
Walk >1 mile	1379	82.2 (80.4-84.1)	28.2 (25.9-30.6)	28.3 (25.7-31.2)	32.7 (29.9-35.1)	36.0 (33.4-38.7)	40.5 (37.7-43.4)	44.2 (41.3-47.3)	<.001	<.001
400-meter Walk test										
Completed	1392	70.2 (67.8-72.8)	78.8 (76.4-81.1)	76.2 (73.8-78.6)	77.7 (75.2-80.3)	78.4 (75.8-81.1)	75.4 (72.5-78.1)	72.2 (68.8-75.0)	.33	.006
Mobility deficit	1310	43.1 (40.2-45.3)	25.2 (22.7-27.4)	26.0 (23.0-28.6)	25.7 (23.2-28.3)	25.6 (22.9-28.2)	27.4 (25.1-30.0)	35.4 (31.8-38.8)	<.001	.02^e^
Time to complete, mean seconds	981	386.4 (382.9-390.2)	348.8 (345.5-352.4)	340.7 (337.1-344.3)	344.8 (341.2-348.3)	344.3 (340.5-347.9)	347.1 (343.8-350.6)	360.8 (356.6-365.3)	<.001	<.001^e^
Fitness proxy, mean										
Resting heart rate, bpm^f^	1149	79.3 (78.6-79.9)	68.0 (67.4-68.6)	69.1 (68.4-69.7)	70.2 (69.6-70.9)	70.2 (69.4-70.8)	70.7 (70.1-71.4)	71.4 (70.6-72.2)	<.001	.003
WOMAC Scores, mean^g^										
Knee physical function^h^	456	47.2 (45.9-48.6)	20.5 (18.8-21.9)	19.8 (18.3-21.2)	21.5 (19.7-23.3)	21.3 (19.7-23.1)	24.7 (22.9-26.4)	25.9 (23.8-28.0)	<.001	<.001
Hip physical function^i^	340	44.6 (43.0-46.3)	18.4 (16.3-20.1)	19.0 (17.0-20.6)	20.0 (18.4-21.8)	21.1 (19.3-22.9)	22.9 (20.8-24.8)	25.5 (23.5-27.8)	<.001	<.001

Abbreviations: bpm, beats per minute; SF-36, Medical Outcomes Study Short-Form 36 Health Survey; WOMAC, Western Ontario and McMaster Universities Osteoarthritis Index.

^a^ Time to complete the 400-meter walk was evaluated among those who started the 400-meter walk; knee function and hip function were evaluated among those with symptoms indicative of osteoarthritis in the respective joint.

^b^ Adjusted for site and age. eTable 4 in the Supplement presents the observed data.

^c^ *P* values from linear trend test are reported if *P* values from quadratic trend were .05 or greater.

^d^ Norm-based methods were used to transform scores (mean [SD], 50 [10]) in the general US population. Higher scores indicate better function.

^e^ Quadratic trend test *P* value.

^f^ Data set to missing at assessments at which participants were taking β-blockers.

^g^ Lower scores indicate less pain and better function on a 0 to 100 point scale.

^h^ Excludes 3 of 459 participants with preoperative symptoms of osteoarthritis in the knee due to missing preoperative knee function score.

^i^ Excludes 7 of 347 participants with preoperative symptoms of osteoarthritis in the hip due to missing preoperative hip function score.

The Figure shows the modeled prevalence of preoperative-to-postoperative CIIs in primary and secondary outcomes by assessment. Descriptive and modeled data are provided in eTable 5 and eTable 6 in the Supplement, respectively. Although the percentage of participants with CIIs in SF-36 bodily pain and physical function scores decreased from 3 years (bodily pain: 50% [95% CI, 48%-53%]; physical function: 75% [95% CI, 73%-77%]) to 7 years after surgery, 43% (95% CI, 40%-46%) still had CIIs in bodily pain, and nearly two-thirds (64% [95% CI, 61%-68%]) did in physical function at 7 years (Figure, A). Half (50% [95% CI, 45%-55%]) of participants who completed the 400-meter walk preoperatively had CIIs in completion time at year 7 (down from 61% [95% CI, 56%-65%] in year 3), and 41% (95% CI, 32%-49%) with a preoperative mobility deficit no longer had one at 7 years (down from 50% [95% CI, 42%-57%] in year 3) (Figure, B). Among participants with preoperative symptoms of osteoarthritis, the 7-year prevalence of CIIs in knee pain and hip pain were 69% (95% CI, 64%-75%) and 65% (95% CI, 58%-72%) respectively (down from 77% [95% CI, 72%-82%] for hip pain in year 3 and 77% [95% CI, 72%-81%] for knee pain in year 3); in knee function and hip function, they were 72% (95% CI, 67%-77%) and 66% (95% CI, 59%-73%), respectively (down from 77% [95% CI, 73%-82%] for knee function in year 3 and 78% [95% CI, 73%-84%] for hip function in year 3) (Figure, C).

**Figure.**
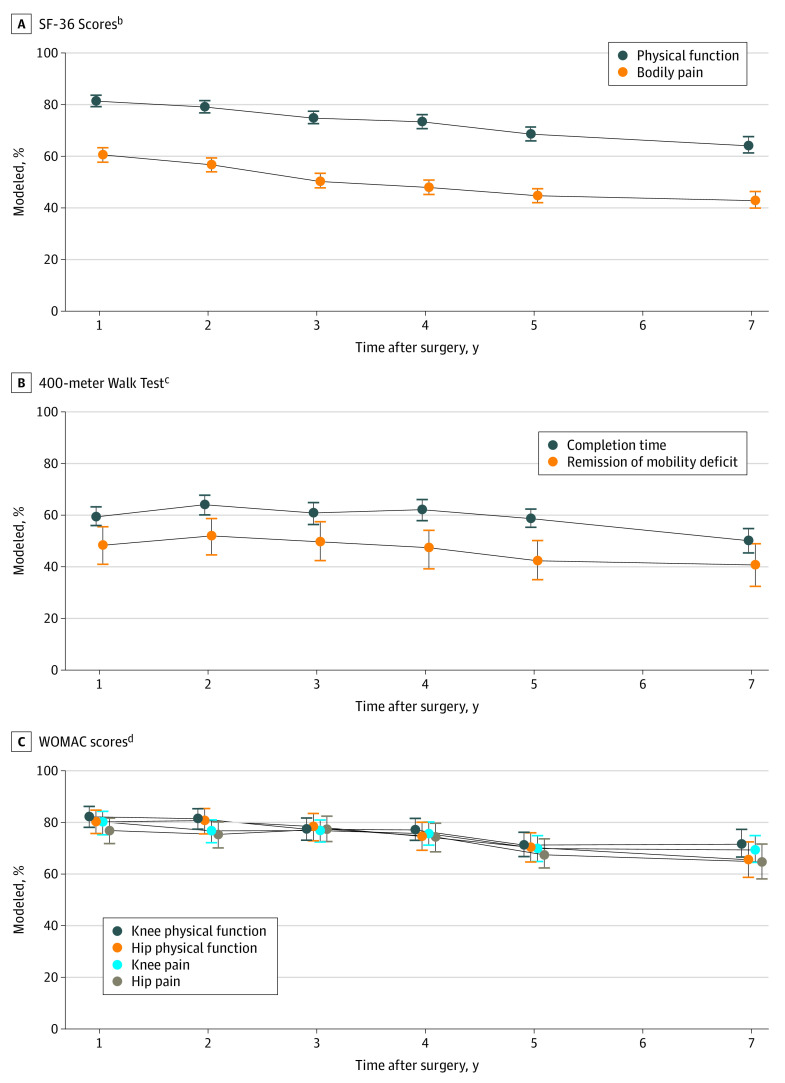
Percentage of Adults with Clinical Important Improvements in Pain and Physical Function Measures by Year Since Roux-en-Y Gastric Bypass (RYGB) or Sleeve Gastrectomy (SG)^a^ All models were adjusted for site and age. Observed and modeled data are reported in eTables 5 and 6 in the Supplement, respectively. SF-36 indicates Medical Outcomes Study 36-Item Short-Form Health Survey; WOMAC, Western Ontario and McMaster Universities Osteoarthritis. ^a^Improvements in bodily pain and physical function were evaluated among the entire sample; 400-meter walk time improvement was evaluated among participants who completed the walk preoperatively and postoperatively; improvements in knee pain and function were evaluated among those with symptoms indicative of osteoarthritis in the knee; improvement in hip pain and function was evaluated among those with preoperative symptoms indicative of osteoarthritis in the hip; and remission of mobility deficit was evaluated among those with a preoperative mobility deficit. ^b^Improvement defined as an increase of at least 5 points on the norm-based scores. ^c^Improvement defined as a decrease in completion time of at least 24 seconds or completed walk in 7 or fewer minutes. ^d^Improvement defined as a decrease of at least 9.7 pain points or 9.3 function points. Overall, 12 of 459 participants with preoperative symptoms of osteoarthritis in the knee and 17 of 347 participants with preoperative symptoms of osteoarthritis in the hip were excluded from analysis of change in pain because their preoperative pain score was less than 9.7 points; 5 of 459 participants with preoperative symptoms of osteoarthritis in the knee and 7 of 347 patients with preoperative symptoms of osteoarthritis in the hip were excluded from analysis of change in function because their preoperative function score was below 9.3 points.

### Work Productivity

Table 3 shows the modeled work productivity measures by assessment; descriptive statistics are provided in eTable 7 in the Supplement. Although absenteeism (ie, missed work due to health), evaluated as any vs some and by percentage, initially decreased (ie, improved) after surgery, absenteeism rebounded by year 3 and then remained fairly stable in years 3 to 7 after surgery. Accordingly, at 7 years, absenteeism was not better than before surgery (eg, participants reporting any at 7 years: 14% [95% CI, 11%-18%]; preoperatively: 16% [95% CI, 13%-19%]). Presenteeism (ie, impaired work due to health), evaluated as any vs some and by percentage, increased in the 3 to 7 years after surgery. However, presenteeism remained lower (ie, better) than preoperative levels (eg, participants reporting any at 7 years: 43% [95% CI, 38%-49%]; preoperatively: 63% [95% CI, 60%-67%]).

**Table 3.** Work Productivity Before and After Roux-en-Y Gastric Bypass and Sleeve Gastrectomy Among 693 Participants^a^

Outcome	Participants, No.	Model-based estimates, % (95% CI)^b^							*P* value	
		Preoperative	Year 1	Year 2	Year 3	Year 4	Year 5	Year 7	Preoperative vs year 7	Years 3 to 7^c^
Absenteeism (any missed work due to health)^d^	644	16.0 (13.0-18.9)	9.2 (7.0-11.8)	10.3 (7.7-12.9)	12.4 (9.6-15.7)	11.9 (9.1-14.7)	11.5 (8.7-14.3)	14.1 (10.7-17.7)	.54	.46
Percentage of work missed due to health^d^										
0	619	81.7 (81.5-86.4)	89.5 (88.1-92.7)	87.8 (87.5-91.5)	85.3 (83.9-90.4)	84.9 (84.5-89.8)	85.6 (85.1-90.0)	84.0 (82.2-87.8)	.81	.28
>0-10		6.4 (5.3-8.5)	2.8 (2.0-5.1)	4.2 (2.4-5.8)	6.1 (5.0-9.1)	3.9 (2.5-6.5)	3.3 (2.0-6.0)	4.2 (1.7-7.1)		
>10-20		4.7 (2.8-6.5)	2.4 (1.9-4.2)	1.9 (0.9-3.9)	4.2 (2.6-6.2)	2.6 (1.3-4.1)	2.6 (1.7-3.6)	4.1 (2.5-7.5)		
>20-30		3.2 (1.4-9.8)	1.1 (0.5-1.8)	2.1 (1.0-4.4)	1.5 (0.3-4.7)	1.0 (0.2-1.7)	2.1 (1.1-3.3)	3.0 (1.6-4.6)		
>30-40		1.1 (0.3-2.7)	1.6 (0.6-4.7)	0.8 (0.2-3.2)	1.2 (0.4-3.4)	2.1 (1.5-3.0)	0.9 (0.1-2.4)	0.7 (0.3-2.4)		
>40-50		0.7 (0.1-2.5)	0.9 (0.6-3.3)	0.5 (0.2-0.9)	0.1 (0.03-0.14)	3.8 (2.1-8.2)	1.3 (0.2-6.4)	0.4 (0.2-3.7)		
>50		2.5 (1.6-4.1)	1.7 (0.7-3.5)	2.8 (1.7-4.3)	1.7 (1.0-2.7)	2.1 (1.2-4.6)	4.3 (2.5-5.6)	3.6 (2.2-7.8)		
Presenteeism (any impairment while working due to health)^e^	615	63.3 (59.5-67.2)	27.1 (23.5-31.0)	31.0 (26.7-34.8)	36.8 (31.8-41.4)	34.3 (30.2-38.3)	39.0 (34.7-43.3)	43.4 (38.3-48.7)	<.001	.01
Percentage of time working impaired due to health^e^										
0	615	36.1 (32.9-40.7)	71.7 (69.8-76.4)	67.6 (65.3-73.4)	62.8 (59.0-67.9)	64.2 (61.5-70.1)	60.8 (57.0-65.9)	56.5 (51.6-62.0)	<.001	.001
10		17.7 (15.5-21.7)	10.9 (8.5-14.7)	11.4 (8.5-15.2)	12.3 (9.6-15.5)	9.7 (7.6-13.2)	11.8 (9.0-15.1)	11.2 (8.2-14.8)		
20		13.7 (11.6-17.4)	6.0 (3.9-8.7)	7.2 (5.0-10.2)	10.6 (7.6-13.8)	8.2 (5.9-11.5)	10.0 (7.3-13.1)	11.2 (8.1-15.7)		
30		11.9 (9.7-15.3)	5.6 (3.7-8.5)	4.0 (2.2-6.3)	5.3 (3.6-7.6)	6.5 (4.4-9.6)	6.9 (4.6-9.7)	6.9 (4.4-10.4)		
40		5.2 (3.5-7.8)	1.8 (0.8-3.5)	2.5 (1.2-4.5)	3.7 (2.2-5.9)	3.4 (1.8-5.9)	3.3 (2.0-5.6)	3.9 (2.1-6.9)		
50		5.3 (3.9-8.4)	1.3 (0.4-2.8)	1.2 (0.3-2.7)	2.0 (0.8-3.9)	2.8 (1.3-5.3)	1.8 (0.7-3.3)	3.8 (1.7-6.4)		
>50		10.1 (7.8-13.6)	2.7 (1.3-4.4)	6.1 (3.8-9.5)	3.3 (1.7-5.6)	5.1 (3.2-8.0)	5.5 (3.6-8.3)	6.5 (3.9-10.3)		

^a^ Among 1491 participants in this report, 796 participants were excluded from work productivity assessment because they did not report being employed at all nonmissing assessments, and 2 were excluded for not answering relevant questions on the Work Productivity and Activity Impairment questionnaire.

^b^ Adjusted for site and age. eTable 7 in the Supplement presents observed data.

^c^ *P* values for linear trends are reported given that all *P* values for quadratic trends were .05 or greater.

^d^ Participants recorded the number of hours worked, missed due to health, and missed due to other reasons. Absenteeism was defined as any work missed due to health. The percentage of work missed due to health was calculated as hours missed from work “because of your health problems” divided by the sum of hours missed for any reason plus hours worked.

^e^ Participants indicated how much their health problems affected their productivity via a rating scale (0 to 10, with 0 indicating “health problems had no effect on my work” and 10, “health problems completely prevented me from working”). A rating greater than 0 indicates presenteeism (ie, health problems affecting work productivity). The response times 10 is assumed to represent a percentage reduction in productive work due to health problems.

Descriptive data among participants who underwent SG are provided in the eTables 8 to 11 in the Supplement. Most estimates and time trends appeared similar to the full sample. However, there were measures that may have indicated less initial improvement (ie, severe walking limitation, mobility aid use) and greater worsening during long-term follow-up (ie, severe walking limitation, completion of 400-meter walk) in the SG subgroup.

## Discussion

In a large US cohort of adults who underwent RYGB or SG, initial preoperative-to-postoperative improvements in pain and physical function decreased over longer-term follow-up through 7 years. Nevertheless, at the 7-year assessment, 41% to 64% experienced preoperative-to-postoperative CIIs in perceived bodily pain and physical function and objectively measured walking capacity, and 65% to 72% with preoperative symptoms indicative of osteoarthritis in the knee or hip experienced CIIs. Furthermore, the long-term reductions in various walking distances limited by health (ie, 1 block, several blocks, >1 mile), time to complete the 400-meter walk, and resting heart rate indicate that, on average, participants experienced durable improvements in walk capacity and fitness.

Some aspects of physical function, such as balance and strength, start to decline by the fifth decade of life, and others, such as walking speed and aerobic endurance, typically decline in the sixth^28,29^; obesity and comorbidities accelerate decline.^22,30^ For example, in a large population-based study that followed adults over 2 decades, while most participants experienced declines in physical function and worsening of bodily pain, compared with those without obesity and no more than 1 metabolic risk factor, those who had obesity and 2 or more metabolic risk factors experienced twice as much decline in physical function and 6 times as much worsening of bodily pain per 10 years.^30^ Thus, while some metrics of physical function (eg, mobility aid use and 400-meter walk completion) had a similar prevalence at 7 years after surgery vs the preoperative period, a lack of worsening might be considered a positive finding given the cohort aged 7 years, to a median age of 54 years, and had severe obesity, often with comorbidities, when follow-up began.

Almost all metrics of pain indicated durable improvements throughout long-term follow-up. However, medication use for back pain was similar to preoperative prevalence in years 4 to 7. In addition, the prevalence of being unable to go to work because of back or leg pain was similar by year 7 vs the preoperative period, mirroring our finding of no long-term improvement in absenteeism due to health. Findings regarding presenteeism were more favorable. Seven years after RYGB or SG, the percentages of participants reporting (1) that back or leg pain interfered with work and (2) any work impairment due to health were lower than in the preoperative period. Likewise, there was a durable reduction in the percentage of work-time impaired due to health. These findings, which may be explained by weight loss or improved physical function,^15^ indicate that modern-day bariatric surgical procedures, on average, improve some aspects of work productivity for at least 7 years. Again, this is an impressive finding giving the countereffect of aging.^31^

Prior to surgery, 30% of participants had undergone ankle, knee, hip, or back surgery. However, bariatric surgical procedures have consistently been shown to improve joint arthroplasty outcomes,^32,33,34^ and it is likely that some participants who needed joint arthroplasty or another orthopedic surgery were ineligible due to a BMI criterion (eg, <40).^35,36^ In this study, we did not assess desire or need for orthopedic surgeries prior to RYGB or SG or the outcomes of such surgeries. However, across 7 years of follow-up, almost one-fifth of participants underwent at least 1 ankle, knee, hip, or back surgery, which may have contributed to reductions in pain and improvements in function.

### Clinical Implications

While this study provides strong evidence for the beneficial associations of RYGB and SG with pain and physical function, it also suggests that not all patients maintain CIIs over long-term follow-up. Some patients likely experience levels of pain and disability following surgery that affect their quality of life and interfere with adopting or maintaining an active lifestyle, especially as time from surgery increases. Thus, clinicians should evaluate postoperative patients who may require additional interventions to improve pain and physical function outcomes. They may be the same patients who experience greater weight regain and declines in their initial improvements in physical and mental health.^14,17,37^ A previous report from the LABS-2 study evaluated the association between preoperative-to-postoperative changes in weight, comorbidities, and mental health with CII in several measures of pain and physical function during the first 3 years of follow-up.^13^ Greater weight loss, not having symptoms of cardiovascular disease in the past year, remission of diabetes, remission of venous edema with ulcerations, and greater improvement in depressive symptoms were independently associated with greater improvements in measures of pain, physical function, or both. Additionally, preoperative-to-postoperative improvements in bodily pain were associated with CII in physical function after controlling for factors related to both pain and function, suggesting effective pain management may help postoperative patients improve their physical function.^13^

### Limitations and Strengths

This study has limitations, including the lack of a nonsurgical control group, which precludes the ability to attribute findings to the surgery itself or ability to compare bariatric surgical procedures. Additionally, the work productivity assessment was restricted to the past week at each time point, and pain measures were imprecise. For example, knee or hip pain may reflect osteoarthritis pain or widespread chronic pain, and bodily pain may reflect all types of pain, including abdominal pain and headaches. Study strengths include the representativeness of the sample to US adults undergoing bariatric surgery in the same timeframe^38^; evaluation of the 2 most common procedures today^9^; inclusion of multiple validated measures of pain, physical function, and work productivity; and annual assessments across a long-term follow-up with relatively high retention.^14^ Additionally, more than 80% of the original LABS-2 cohort who underwent RYGB and SG completed pain and physical function assessments 5 or more years after surgery, and within this sample, analyses controlled for preoperative factors associated with missing follow-up data.^13^

## Conclusions

In this large US cohort of adults who underwent RYGB or SG for obesity, despite declines in preoperative-to-postoperative improvements across follow-up, CIIs in perceived bodily and joint-specific pain and in self-reported and objectively measured physical function ranged from 41% to 72%, depending on the measure and subgroup, 7 years after surgery, suggesting RYGB and SG are often associated with long-term improvements in pain and physical function.

## Notes

Supplement. **eAppendix.** Supplementary Methods **eTable 1.** A Comparison of LABS-2 Participants Who Underwent RYGB or SG Included vs Excluded From the Analysis Sample Due to Missing Data **eTable 2.** Descriptive Statistics of Age, BMI, and Joint Surgeries by Year Since RYGB or SG (n=1491) **eTable 3.** Descriptive Statistics of Pain Measures by Time in Relation to RYGB or SG (n=1491) **eTable 4.** Descriptive Statistics of Physical Function Measures by Time in Relation to RYGB or SG (n=1491) **eTable 5.** Observed Percentage of Patients With Clinically Important Improvements in Pain and Physical Function by Year Since RYGB and SG **eTable 6.** Modeled Percentage of Patients With Clinically Important Improvements in Pain and Physical Function by Year Since RYGB and SG **eTable 7.** Descriptive Statistics of Employment and Work Productivity Measures by Time in Relation to RYGB or SG (n=695) **eTable 8.** Descriptive Statistics of Pain Measures by Time in Relation to SG (n=51) **eTable 9.** Descriptive Statistics of Physical Function Measures by Time in Relation to SG (n=51) **eTable 10.** Observed Percentage of Patients with Clinically Important Improvements in Pain and Physical Function by Year Since SG **eTable 11.** Descriptive Statistics of Employment and Work Productivity Measures by Time in Relation to SG (n=23)
