# Supplementary material for: A 7-Year Study of the Durability of Improvements in Pain, Physical Function, and Work Productivity After Roux-en-Y Gastric Bypass and Sleeve Gastrectomy
Source: JAMA Netw Open. 2022 Sep 14;5(9):e2231593. doi: 10.1001/jamanetworkopen.2022.31593 (PMC9475385; doi:10.1001/jamanetworkopen.2022.31593)
Supplement: Supplement. — eAppendix. Supplementary Methods eTable 1. A Comparison of LABS-2 Participants Who Underwent RYGB or SG Included vs Excluded From the Analysis Sample Due to Missing Data eTable 2. Descriptive Statistics of Age, BMI, and Joint Surgeries by Year Since RYGB or SG (n=1491) eTable 3. Descriptive Statistics of Pain Measures by Time in Relation to RYGB or SG (n=1491) eTable 4. Descriptive Statistics of Physical Function Measures by Time in Relation to RYGB or SG (n=1491) eTable 5. Observed Percentage of Patients With Clinically Important Improvements in Pain and Physical Function by Year Since RYGB and SG eTable 6. Modeled Percentage of Patients With Clinically Important Improvements in Pain and Physical Function by Year Since RYGB and SG eTable 7. Descriptive Statistics of Employment and Work Productivity Measures by Time in Relation to RYGB or SG (n=695) eTable 8. Descriptive Statistics of Pain Measures by Time in Relation to SG (n=51) eTable 9. Descriptive Statistics of Physical Function Measures by Time in Relation to SG (n=51) eTable 10. Observed Percentage of Patients with Clinically Important Improvements in Pain and Physical Function by Year Since SG eTable 11. Descriptive Statistics of Employment and Work Productivity Measures by Time in Relation to SG (n=23) [file jamanetwopen-e2231593-s001.pdf]

Supplemental Online Content

King WC, Hinerman AS, White GE. A 7-year study of the durability of improvements in pain, physical function, and work productivity after Roux-en-Y gastric bypass and sleeve gastrectomy. *JAMA Netw Open*. 2022;5(9):e2231593. doi:10.1001/jamanetworkopen.2022.31593

eAppendix. Supplementary Methods

eTable 1. A Comparison of LABS-2 Participants Who Underwent RYGB or SG Included vs Excluded From the Analysis Sample Due to Missing Data

eTable 2. Descriptive Statistics of Age, BMI, and Joint Surgeries by Year Since RYGB or SG (n=1491)

eTable 3. Descriptive Statistics of Pain Measures by Time in Relation to RYGB or SG (n=1491)

eTable 4. Descriptive Statistics of Physical Function Measures by Time in Relation to RYGB or SG (n=1491)

eTable 5. Observed Percentage of Patients With Clinically Important Improvements in Pain and Physical Function by Year Since RYGB and SG

eTable 6. Modeled Percentage of Patients With Clinically Important Improvements in Pain and Physical Function by Year Since RYGB and SG

eTable 7. Descriptive Statistics of Employment and Work Productivity Measures by Time in Relation to RYGB or SG (n=695)

eTable 8. Descriptive Statistics of Pain Measures by Time in Relation to SG (n=51)

eTable 9. Descriptive Statistics of Physical Function Measures by Time in Relation to SG (n=51)

eTable 10. Observed Percentage of Patients With Clinically Important Improvements in Pain and Physical Function by Year Since SG

eTable 11. Descriptive Statistics of Employment and Work Productivity Measures by Time in Relation to SG (n=23)

This supplemental material has been provided by the authors to give readers additional information about their work.

© 2022 King WC et al. *JAMA Network Open.*

eAppendix 1. Supplementary Methods

Pain and Physical Function. The Medical Outcomes Study 36-item Short-Form Health Survey (SF- 36) is a generic measure of functional health and well-being with proven validity, reliability and sensitivity to change[1]. Two physical domains scores were examined- bodily pain, comprised of two items which assess the magnitude of bodily pain and how much it interferes with activities, and physical function, comprised of ten items which assess the impact of health problems related to the ability to participate in physical activities. Norm-based methods were used to transform the scores to a mean of 50 and standard deviations of 10 in the general U.S. population[2]. Higher SF-36 scores imply less pain or better function. The three items specific to walking limitations (health limits ability to walk 1 block, several blocks and walking more than a mile) were also examined.

The Western Ontario and McMaster Universities Osteoarthritis index (WOMAC) measures symptoms of hip and knee osteoarthritis. It has demonstrated reliably and validity and is sensitive to pre- to post-intervention changes[3]. For each joint, participants were categorized as having symptoms indicative of osteoarthritis if they choose a severe or extreme rating on ≥1 item[4]. Among those with such symptoms, two scores per joint were examined: pain, comprised of five items that assess pain level during various activities; and function, comprised of 17 items which assess difficulty performing various activities. Scores were transformed to a 0-100 scale[5]. Lower WOMAC scores imply less pain and better function.

Participants reported back, hip, knee and ankle surgery (lifetime history at preoperative; past year at annual assessments), use of prescription or over-the-counter pain medication for back or leg (hip, knee or ankle) pain, respectively, in the past week, how much back or leg pain interfered with work (work outside the home and house work) in the past 4 weeks, inability to go to work or school due to back or leg pain in the past 4 weeks, level of dissatisfaction/satisfaction with current back or leg pain, as well as severe walking limitation (self-reported inability to walk 200 feet [61 m] without assistance) and mobility aid use[6].

The 400-meter Long Distance Corridor Walk (400-m walk test) was used to objectively measure walking capacity[7,8]. To minimize risk, participants were instructed to walk the 400 m course “at your usual pace” and wore a heart rate monitor. At the end of the walk, participants were asked if they had experienced back, leg (hip, knee or foot) pain during the 400-m walk test. Testing could be terminated prior to completing 400 m for safety reasons. Participants were ineligible to attempt the 400-m walk test if they had a contraindication to exertion, had any of several cardiovascular risk factors, needed a mobility aid other than a straight cane, or reported feeling unsafe. Participants were categorized as having a mobility deficit if they met the 400-m walk test exclusion criteria or stopping criteria, or exceeded 7 minutes to walk 400 m, which equates to a cardiorespiratory fitness level of <12 mL oxygen/kg/min, the minimal level deemed necessary to safely cross a traffic intersection[8].

Heart rate, measured after a minimum of 5 minutes of seated rest, was used as a proxy for cardiovascular fitness.

Work Productivity. The Work Productivity and Activity Impairment (WPAI) questionnaire used across various occupations and disease areas to assess employee productivity related to health[9]. Questions are asked in reference to the past week. Those who indicate working for pay record the number of hours worked, missed due to health, and missed due to other reasons (e.g., vacation). The percentage of work missed due to health is calculated as hours missed from work “because of your health problems,” divided by the sum of hours missed for any reason plus hours worked. Absenteeism is defined as any work missed due to health. Participants indicate how much their health problems affected their productivity via a rating scale (0 to 10; 0 = “health problems had

© 2022 King WC et al. *JAMA Network Open.*

no effect on my work" to 10 = "health problems completely prevented me from working"). The response times 10 is assumed to represent a percentage reduction in productive work due to health problems (e.g., a respondent reporting a value of 3 is assumed to have a 30% reduction in productive work). Presenteeism is defined as health problems affecting work productivity.

Anthropometrics, sociodemographics and health indicators. Anthropometric measurements followed standardized protocols[10]. Sociodemographics and smoking status were self-reported. Race and ethnicity were collected as descriptors of the study sample, to address generalizability of the study findings. The variable race/ethnicity was based on self-report of race and ethnicity. If race or ethnicity were missing, race/ethnicity was set to missing. Additionally, race was set to missing for participants who did not self-report their race as one or more of the following options- white/Caucasian, black/African-American, Asian, American Indian/Alaska Native, Native Hawaiian/other Pacific Islander. Comorbidities (diabetes, cardiovascular disease, asthma, sleep apnea and venous edema with ulcerations) were determined using a combination of laboratory values, physical examination measures, participant reported medication use, comorbidity diagnoses from healthcare providers and medical records review[11].

Reference List

1. Frendl DM, Ware JE, Jr. Patient-reported functional health and well-being outcomes with drug therapy- a systematic review of randomized trials using the SF-36 health survey. *Med Care* 2014; 52(5)-439-445.

2. Ware JE, Jr., Kosinski M, Keller SK. SF-36® physical and mental health summary scales- a user's manual. Boston, MA- The Health Institute, 1994.

3. McConnell S, Kolopack P, Davis AM. The Western Ontario and McMaster Universities Osteoarthritis Index (WOMAC)- a review of its utility and measurement properties. *Arthritis Care and Research* 2001; 45(5)-453-461.

4. Jinks C, Jordan K, Croft P. Measuring the population impact of knee pain and disability with the Western Ontario and McMaster Universities Osteoarthritis Index (WOMAC). Pain. 2002;100(1-2):55-64

5. Bellamy N. The WOMAC Knee and Hip Osteoarthritis Indices- development, validation, globalization and influence on the development of the AUSCAN Hand Osteoarthritis Indices. *Clin Exp Rheumatol* 2005; 23(5 Suppl 39)-S148-S153.

6. Belle SH, Berk PD, Courcoulas AP, Flum DR, Miles CW, Mitchell JE et al. Safety and efficacy of bariatric surgery- Longitudinal Assessment of Bariatric Surgery. *Surg Obes Relat Dis* 2007; 3(2)-116-126.

7. Lange-Maia BS, Newman AB, Strotmeyer ES, Harris TB, Caserotti P, Glynn NW. Performance on fast- and usual-paced 400-m walk tests in older adults- are they comparable? *Aging Clin Exp Res* 2015; 27(3)-309-314.

8. Rolland YM, Cesari M, Miller ME, Penninx BW, Atkinson HH, Pahor M. Reliability of the 400-m usual-pace walk test as an assessment of mobility limitation in older adults. *J Am Geriatr Soc* 2004; 52(6)-972-976.

9. Reilly MC, Zbrozek AS, Dukes EM. The validity and reproducibility of a work productivity and activity impairment instrument. *Pharmacoeconomics* 1993 ;4:353-365.

10. Courcoulas AP, Christian NJ, Belle SH, Berk PD, Flum DR, Garcia L et al. Weight change and health outcomes at 3 years after bariatric surgery among individuals with severe obesity. *JAMA* 2013; 310(22)-2416-2425.

11. Belle SH, Berk PD, Chapman WH, et al; LABS Consortium. Preoperative characteristics of participants in the Longitudinal Assessment of Bariatric Surgery-2 (LABS-2) study. Surg Obes Relat Dis. 2013;9(6)-926-935.

© 2022 King WC et al. *JAMA Network Open.*

eTable 1. A Comparison of LABS-2 Participants Who Underwent RYGB or SG Included vs Excluded From the Analysis Sample Due to Missing Data

	No. (%)[a]		
	**Excluded**	**Included**	
	**n=338**	**n=1491**	**P-value**
Female	258 (76.3)	1194 (80.1)	0.12
Age, years			<0.001
Median (25th-75th)	41 (35-48)	47 (38-55)	
Range	19-68	19-75	
Age, years			<0.001
18 - <30	42 (12.4)	125 (8.4)	
30 - <40	109 (32.2)	341 (22.9)	
40 - <50	118 (34.9)	412 (27.6)	
50 - <60	56 (16.6)	443 (29.7)	
60 - <70	13 (3.8)	162 (10.9)	
≥70	0 (0.0)	8 (0.5)	
Race/ethnicity	n=331	n=1477	0.74
Hispanic	16 (4.8)	59 (4.0)	
Non-Hispanic Black	35 (10.6)	164 (11.1)	
Non-Hispanic White	272 (82.2)	1205 (81.6)	
Other	8 (2.4)	49 (3.3)	
Work status and productivity			
Work for pay, No./total (%)	207/305 (67.9%)	953/1377 (69.2%)	0.65
Absenteeism[b]	31/201 (15.4%)	179/930 (19.2%)	0.21
Presenteeism[c]	131/190 (68.9%)	582/890 (65.4%)	0.35
Household income, US $	n=298	n=1344	0.17
less than $25,000	62 (20.8)	270 (20.1)	
$25,000-$49,000	81 (27.2)	377 (28.1)	
$50,000-$74,999	80 (26.8)	305 (22.7)	
$75,000-$99,999	42 (14.1)	204 (15.2)	
$100,000 or greater	33 (11.1)	188 (14.0)	
Body mass index, kg/m^2			0.12
Median (25th-75th)	47.7 (42.8-53.0)	46.5 (42.4-52.3)	
Range	33.9-77.2	33.7-94.3	
Current or recent smoker, No./total (%)	69/336 (20.5)	195/1489 (13.1)	<0.001
Comorbidity and surgical history, No./total (%)			
Cardiovascular disease	20/329 (6.1)	127/1460 (8.7)	0.12
Stroke	2/337 (0.6)	16/1489 (1.1)	0.42
Diabetes	106/328 (32.3)	526/1464 (35.9)	0.22
Sleep apnea	178/337 (52.8)	764/1490 (51.3)	0.61
Asthma	78/327 (23.9)	386/1457 (26.5)	0.33
Venous edema with ulcerations	23/337 (6.8)	125/1490 (8.4)	0.34
Back surgery	20/303 (6.6)	111/1367 (8.1)	0.37
Hip surgery	5/302 (1.7)	47/1365 (3.4)	0.11
Knee surgery	51/303 (16.8)	275/1368 (20.1)	0.19
Ankle surgery	17/303 (5.6)	76/1366 (5.6)	0.97
Walking status			
Severe walking limitation, No./total (%)	23/306 (7.5)	95/1346 (7.1)	0.78
Completed the 400-m walk test, No./total (%)	210/315 (66.7)	982/1392 (70.5)	0.18
400-m walk time, seconds[d]	n=210	n=981	0.56
Median (25th-75th)	380.1 (337.5-419.1)	373.5 (337.3-416.5)	
Range	202.0-633.2	238.2-802.9	
Mobility disability, No./total (%)	125/284 (44.0)	557/1310 (42.5)	0.64
SF-36 scores			
Bodily pain	n=306	n=1378	0.62
Median (25th-75th)	39.6 (31.5-44.3)	37.7 (31.5-48.5)	
Range	18.0-60.4	18.0-60.4	
Physical function	n=304	n=1382	0.70
Median (25th-75th)	37.1 (28.4-45.8)	34.9 (26.2-45.8)	
Range	13.1-56.8	13.1-56.8	
WOMAC			
Symptoms of osteoarthritis of the knee	91/261 (34.9)	459/1088 (42.2)	0.03
Knee pain score[e]	n=91	n=455	0.60
Median (25th-75th)	50 (35-60)	45 (30-60)	
Range	15-90	0-100	
Knee function score[e]	n=89	n=456	0.25

© 2022 King WC et al. *JAMA Network Open.*

Median (25th-75th)	48.5 (39-63)	47 (35-59)	
Range	3-99	6-100	
Continued next page			
	**Excluded**	**Included**	
	**n=338**	**n=1491**	**P-value**
Symptoms of osteoarthritis of the hip	69/260 (26.5)	347/1089 (31.9)	0.09
Hip pain score[f]	n=68	n=347	0.25
Median (25th-75th)	50 (35-63)	50 (30-60)	
Range	15-100	0-100	
Hip function score[f]	n=69	n=340	0.06
Median (25th-75th)	50 (40-63)	46 (34-57)	
Range	9-86	0-100	

Abbreviations: LABS, Longitudinal Assessment of Bariatric Surgery; RYGB, Roux-en-Y gastric bypass; SF-36, Medical Outcomes Study Short-Form 36 Health Survey; SG, sleeve gastrectomy; WOMAC, Western Ontario and McMaster Universities Osteoarthritis Index.

[a]Data are reported as No. (%) unless otherwise indicated.

[b]Missed work due to health in past 7 days. Reported among those who work for pay.

[c]Impaired while working due to health in past 7 days. Reported among those who work for pay.

[d] Reported among participants who completed the 400-m walk test.

[e] Reported among participants with preoperative symptoms of osteoarthritis of the knee.

[f] Reported among participants with preoperative symptoms of osteoarthritis of the hip.

© 2022 King WC et al. *JAMA Network Open.*

eTable 2. Descriptive Statistics of Age, BMI, and Joint Surgeries by Year Since RYGB or SG (n=1491)

	Year 1	Year 2	Year 3	Year 4	Year 5	Year 7
Age, years	n=1491	n=1491	n=1491	n=1491	n=1491	n=1491
Median (25th-75th)	48 (39-56)	49 (40-57)	50 (41-58)	51 (42-59)	52 (43-60)	54 (45-62)
Age, years, n (%)	n=1491	n=1491	n=1491	n=1491	n=1491	n=1491
18 - <30	90 (6.0)	76 (5.1)	64 (4.3)	51 (3.4)	39 (2.6)	27 (1.8)
30 - <40	303 (20.3)	275 (18.4)	254 (17.0)	234 (15.7)	208 (14.0)	175 (11.7)
40 - <50	415 (27.8)	411 (27.6)	394 (26.4)	386 (25.9)	381 (25.6)	370 (24.8)
50 - <60	463 (31.1)	457 (30.7)	466 (31.3)	457 (30.7)	455 (30.5)	436 (29.2)
60 - <70	207 (13.9)	253 (17.0)	281 (18.8)	320 (21.5)	352 (23.6)	397 (26.6)
≥70	13 (0.9)	19 (1.3)	32 (2.1)	43 (2.9)	56 (3.8)	86 (5.8)
Work for pay, No./total (%)	829/1217 (68.1%)	741/1114 (66.5%)	713/1092 (65.3%)	698/1095 (63.7%)	717/1183 (60.6%)	514/838 (61.3%)
Work for pay among non-retired participants, No./total (%)	829/1130 (73.4%)	741/1012 (73.2%)	713/984 (72.5%)	698/968 (72.1%)	717/1034 (69.3%)	514/729 (70.5%)
Body mass index, kg/m^2	n=1438	n=1394	n=1384	n=1397	n=1426	n=984
Median (25th-75th)	30.9 (27.3-35.3)	30.6 (27.0-35.1)	31.7 (27.8-36.2)	32.6 (28.6-37.5)	33.1 (28.9-38.3)	33.5 (29.4-38.8)
Weight loss, % of pre-operative weight	n=1438	n=1394	n=1384	n=1397	n=1426	n=984
Median (25th-75th)	33.8 (28.3-39.0)	34.0 (27.5-40.3)	31.5 (24.8-38.2)	29.6 (22.7-37.0)	28.9 (21.7-36.2)	27.8 (20.1-35.6)
Surgery in past year[a], No./total No. (%)	51/1209 (4.2)	74/1105 (6.7)	76/1084 (7.0)	87/1098 (7.9)	77/1180 (6.5)	51/835 (6.1)
Ankle	11/1207 (0.9)	11/1103 (1.0)	14/1080 (1.3)	15/1095 (1.4)	10/1177 (0.8)	7/835 (0.8)
Back	15/1207 (1.2)	24/1105 (2.2)	29/1086 (2.7)	37/1100 (3.4)	30/1180 (2.5)	16/836 (1.9)
Hip	10/1206 (0.8)	14/1102 (1.3)	18/1081 (1.7)	23/1097 (2.1)	16/1177 (1.4)	8/837 (1.0)
Knee	24/1207 (2.0)	45/1103 (4.1)	41/1082 (3.8)	47/1095 (4.3)	44/1180 (3.7)	27/836 (3.2)

Abbreviations: RYGB, Roux-en-Y gastric bypass; SG, sleeve gastrectomy.
[a]Among participants reporting whether they had past-year surgeries at ≥4 postoperative assessments (N=1112), 23% (n=252) underwent back, hip, knee and/or ankle surgery during follow-up: 5% (n=51) ankle surgery, 9% (n=97) back surgery, 6% (n=69) hip surgery and 14% (n=160) knee surgery.

© 2022 King WC et al. *JAMA Network Open.*

eTable 3. Descriptive Statistics of Pain Measures by Time in Relation to RYGB or SG (n=1491[a])

	No./total No. (%)[a]						
	**Preoperative**	**Year 1**	**Year 2**	**Year 3**	**Year 4**	**Year 5**	**Year 7**
**SF-36 Score**[b]							
Bodily pain	n=1378	n=1215	n=1119	n=1089	n=1100	n=1179	n=835
Median (25th- 75th percentile)	37.7 (31.5-48.5)	49.4 (39.6-53.6)	48.5 (35.8-53.6)	44.3 (35.4-53.6)	44.3 (35.4-53.6)	44.3 (35.4-53.6)	44.3 (35.4-53.6)
**Back or Leg Pain**							
Medication for back pain, past week	480/1359 (35.3)	368/1256 (29.3)	334/1134 (29.5)	349/1113 (31.4)	399/1140 (35.0)	451/1244 (36.3)	326/881 (37.0)
Medication for leg pain, past week	567/1358 (41.8)	342/1253 (27.3)	329/1132 (29.1)	351/1112 (31.6)	404/1132 (35.7)	439/1240 (35.4)	338/877 (38.5)
Back or leg pain interfered with work (outside the home or house work), past 4 weeks, n (%)	n=1369	n=1212	n=1111	n=1089	n=1103	n=1180	n=839
Not at all	651 (47.6)	875 (72.2)	823 (74.1)	786 (72.2)	782 (70.9)	805 (68.2)	548 (65.3)
A little bit	199 (14.5)	128 (10.6)	82 (7.4)	86 (7.9)	99 (9.0)	107 (9.1)	89 (10.6)
Moderately	205 (15.0)	92 (7.6)	74 (6.7)	96 (8.8)	83 (7.5)	100 (8.5)	76 (9.1)
Quite a bit	206 (15.0)	76 (6.3)	85 (7.7)	68 (6.2)	98 (8.9)	113 (9.6)	80 (9.5)
Extremely	108 (7.9)	41 (3.4)	47 (4.2)	53 (4.9)	41 (3.7)	55 (4.7)	46 (5.5)
Could not go to work or school due to back or leg pain, past 4 weeks	97/1360 (7.1)	47/1206 (3.9)	39/1105 (3.5)	37/1080 (3.4)	46/1097 (4.2)	58/1170 (5.0)	52/831 (6.3)
Feelings regarding back or leg pain, n (%)	n=1367	n=1210	n=1109	n=1085	n=1101	n=1181	n=838
Very dissatisfied	357 (26.1)	91 (7.5)	82 (7.4)	92 (8.5)	105 (9.5)	117 (9.9)	99 (11.8)
Dissatisfied	215 (15.7)	94 (7.8)	81 (7.3)	75 (6.9)	78 (7.1)	101 (8.6)	57 (6.8)
Somewhat dissatisfied	123 (9.0)	91 (7.5)	82 (7.4)	93 (8.6)	92 (8.4)	114 (9.7)	83 (9.9)
Neither satisfied or dissatisfied	39 (2.9)	51 (4.2)	44 (4.0)	43 (4.0)	49 (4.5)	50 (4.2)	44 (5.3)
Somewhat satisfied	13 (1.0)	29 (2.4)	19 (1.7)	26 (2.4)	14 (1.3)	18 (1.5)	23 (2.7)
Satisfied	12 (0.9)	38 (3.1)	28 (2.5)	23 (2.1)	29 (2.6)	27 (2.3)	19 (2.3)
Very satisfied	608 (44.5)	816 (67.4)	773 (69.7)	733 (67.6)	734 (66.7)	754 (63.8)	513 (61.2)
Back pain during 400-m walk[c]	190/1033 (18.4)	67/916 (7.3)	48/756 (6.3)	63/750 (8.4)	74/759 (9.7)	54/766 (7.0)	50/517 (9.7)
Leg pain during 400-m walk[c]	434/1033 (42.0)	180/917 (19.6)	149/756 (19.7)	162/750 (21.6)	177/759 (23.3)	180/766 (23.5)	138/518 (26.6)
**WOMAC Scores**[d]							
Knee pain[e]	n=455	n=346	n=359	n=339	n=345	n=365	n=245
Median (25th-75th percentile)	45.0 (30.0-60.0)	15.0 (5.0-35.0)	15.0 (0.0-40.0)	20.0 (0.0-40.0)	20.0 (0.0-40.0)	25.0 (5.0-45.0)	25.0 (5.0-45.0)
Hip pain[f]	n=347	n=263	n=271	n=257	n=262	n=272	n=186
Median (25th-75th percentile)	50.0 (30.0-60.0)	15.0 (0.0-45.0)	15.0 (0.0-45.0)	15.0 (0.0-40.0)	20.0 (0.0-45.0)	20.0 (0.0-50.0)	25.0 (5.0-45.0)

Abbreviations: RYGB Roux-en-Y gastric bypass; SF-36, Medical Outcomes Study Short-Form 36 Health Survey; SG, sleeve gastrectomy; WOMAC, Western Ontario and McMaster Universities Osteoarthritis Index.

[a] Unless otherwise indicated.

[b] Norm-based methods were used to transform scores (mean [SD], 50 [10]) in the general US population. Higher scores indicate less pain.

[c] N=1033; excludes participants who did not start the 400-m walk preoperative.

[d] Lower scores indicate less pain on a 0- to 100-point scale.

[e] N=455; excludes participants who did not have preoperative symptoms of osteoarthritis of the knee. Also excludes 4 participants missing preoperative knee pain score.

[f] N=347; excludes participants who did not have preoperative symptoms of osteoarthritis of the hip.

© 2022 King WC et al. *JAMA Network Open.*

eTable 4. Descriptive Statistics of Physical Function Measures by Time in Relation to RYGB or SG (n=1491[a])

	No./total No. (%)[a]						
	**Preoperative**	**Year 1**	**Year 2**	**Year 3**	**Year 4**	**Year 5**	**Year 7**
**SF-36 Score[b]**							
Physical function	n=1382	n=1218	n=1119	n=1095	n=1098	n=1182	n=837
Median (25th-75th percentile)	34.9 (26.2-45.8)	54.6 (45.8-56.8)	54.6 (45.8-56.8)	52.4 (43.7-56.8)	52.4 (41.5-56.8)	50.2 (39.3-56.8)	50.2 (37.1-54.6)
**Self-Reported Walking**							
Severe walking limitation	95/1346 (7.1)	53/1143 (4.6)	48/1040 (4.6)	58/1041 (5.6)	64/1061 (6.0)	83/1142 (7.3)	53/793 (6.7)
Mobility aid use	211/1348 (15.7)	118/1160 (10.2)	115/1008 (11.4)	119/938 (12.7)	124/945 (13.1)	144/993 (14.5)	123/670 (18.4)
Health Limits Ability to:							
Walk 1 block	569/1373 (41.4)	117/1213 (9.6)	128/1116 (11.5)	129/1090 (11.8)	157/1093 (14.4)	200/1176 (17.0)	165/835 (19.8)
Walk several blocks	930/1379 (67.4)	216/1217 (17.7)	213/1119 (19.0)	240/1089 (22.0)	260/1091 (23.8)	330/1177 (28.0)	253/833 (30.4)
Walk >1 mile	1130/1379 (81.9)	350/1215 (28.8)	327/1118 (29.2)	356/1091 (32.6)	396/1094 (36.2)	479/1179 (40.6)	358/833 (43.0)
**400-m Walk Test**							
Completed	982/1392 (70.5)	914/1143 (80.0)	752/975 (77.1)	743/946 (78.5)	757/960 (78.9)	762/1000 (76.2)	514/684 (75.1)
Mobility deficit	557/1310 (42.5)	271/1085 (25.0)	246/937 (26.3)	225/900 (25.0)	221/895 (24.7)	238/926 (25.7)	199/638 (31.2)
Time to complete, seconds[c]	n=981	n=697	n=558	n=527	n=538	n=542	n=410
Median	373.5	338.0	333.8	336.5	335.0	335.5	347.4
(25th-75th percentile)	(337.3-416.5)	(309.6-374.8)	(300.8-367.6)	(306.0-371.5)	(306.4-372.1)	(306.4-369.5)	(317.3-379.8)
**Fitness Proxy**							
Resting heart rate, bpm[d]	n=1149	n=1179	n=1070	n=1035	n=1024	n=1057	n=716
	80 (72-87)	67 (60-75)	67.5 (60-77)	69 (62-77)	69 (62-78)	70 (62-78)	71 (63-79)
**WOMAC Scores[e]**							
Knee function[f]	n=456	n=342	n=357	n=334	n=344	n=362	n=246
Median (25th-75th percentile)	47.0 (35.3-58.8)	15.3 (2.9-33.8)	13.2 (2.9-36.8)	16.2 (2.9-33.8)	16.2 (1.5-38.2)	23.5 (4.4-42.6)	25.0 (4.4-41.2)
Hip function[g]	n=340	n=262	n=271	n=255	n=262	n=273	n=185
Median (25th-75th percentile)	45.6 (33.8-57.3)	10.3 (0.0-35.3)	8.8 (0.0-36.8)	11.8 (0.0-32.6)	15.1 (0.0-38.2)	16.2 (1.5-44.1)	26.5 (4.4-41.2)

Abbreviations: RYGB Roux-en-Y gastric bypass; SF-36, Medical Outcomes Study Short-Form 36 Health Survey; SG, sleeve gastrectomy; WOMAC, Western Ontario and McMaster Universities osteoarthritis index; bpm, beats per minute.

[a] Unless otherwise indicated.

[b] Norm-based methods were used to transform SF-36 scores to a mean of 50 and standard deviations of 10 in the general U.S. population. Higher scores imply better function.

[c] N=981; excludes 411 of 1392 participants who did not complete the 400-m walk preoperative.

[d] Data set to missing at assessments at which participants were taking B-blockers.

[e] Lower scores indicate less pain and better function on a 0- to 100-point scale.

[f] N=456; excludes participants who did not preoperative symptoms of osteoarthritis of the knee. Also excludes 3 participants missing preoperative knee function score.

[g] N=340; excludes participants who did not have preoperative symptoms of osteoarthritis of the hip. Also excludes 7 participants missing preoperative hip function pain score.

© 2022 King WC et al. *JAMA Network Open.*

eTable 5. Observed Percentage of Patients with Clinically Important Improvements in Pain and Physical Function [a] by Year Since RYGB and SG

	No./total No. (%)					
	**Year 1**	**Year 2**	**Year 3**	**Year 4**	**Year 5**	**Year 7**
SF-36[b]						
Bodily Pain	694/1143 (60.7)	598/1051 (56.9)	518/1021 (50.7)	499/1027 (48.6)	491/1101 (44.6)	348/796 (43.7)
Physical function	938/1151 (81.5)	833/1054 (79.0)	768/1031 (74.5)	755/1029 (73.4)	757/1107 (68.4)	519/800 (64.9)
400-m walk time[c]	420/696 (60.3)	358/557 (64.3)	319/526 (60.6)	334/538 (62.1)	312/542 (57.6)	200/410 (48.8)
Mobility deficit remission	83/172 (48.3)	78/157 (49.7)	64/139 (46.0)	63/142 (44.4)	59/146 (40.4)	42/105 (40.0)
WOMAC						
Knee pain[d]	268/334 (80.2)	264/344 (76.7)	250/326 (76.7)	253/333 (76.0)	247/353 (70.0)	164/236 (69.5)
Hip pain[d]	193/251 (76.9)	193/257 (75.1)	189/244 (77.5)	190/252 (75.4)	178/261 (68.2)	115/180 (63.9)
Knee function[e]	277/337 (82.2)	288/353 (81.6)	257/329 (78.1)	264/339 (77.9)	252/355 (71.0)	172/242 (71.1)
Hip function[e]	201/251 (80.1)	210/259 (81.1)	193/244 (79.1)	192/253 (75.9)	186/263 (70.7)	116/179 (64.8)

Abbreviations: RYGB Roux-en-Y gastric bypass; SF-36, Medical Outcomes Study Short-Form 36 Health Survey; SG, sleeve gastrectomy; WOMAC, Western Ontario and McMaster Universities Osteoarthritis Index.

[a] Improvements in bodily pain and physical function were evaluated among the entire cohort; 400-m walk time improvement was evaluated among participants who completed the walk preoperative; improvement in knee or hip pain and function were evaluated among those with severe pain or disability preoperative; remission of mobility deficit was evaluated among those with a mobility deficit preoperative.

[b] Norm-based score improvement (increase) of at least 5 points.

[c] Improvement (decrease) of at least 24 seconds.

[d] Score improvement (decrease) of at least 9.7 pain points. Twelve of 459 participants with preoperative symptoms of osteoarthritis of the knee and 17 of 347 participants with preoperative symptoms of osteoarthritis of the hip were excluded from analysis because their preoperative pain score was less than 9.7 points.

[e] Scores improvement (decrease) of at least 9.3 function points. Five of 459 participants with preoperative symptoms of osteoarthritis of the knee and 7 of 347 participants with preoperative symptoms of osteoarthritis of the hip were excluded from analysis because their preoperative function score was less than 9.3 points.

© 2022 King WC et al. *JAMA Network Open.*

eTable 6. Modeled Percentage of Patients with Clinically Important Improvements in Pain and Physical Function[a] by Year Since RYGB and SG

		Model-Based Estimates, % (95% CI)[b]						*P* Value
	**N**	**Year 1**	**Year 2**	**Year 3**	**Year 4**	**Year 5**	**Year 7**	**Years 3 to 7[c]**
SF-36[d]								
Bodily pain	1143	60.6 (57.7-63.3)	56.8 (54.0-59.4)	50.3 (47.8-53.4)	48.0 (45.2-50.9)	44.7 (42.1-47.4)	42.9 (39.9-46.4)	<.001
Physical function	1151	81.3 (79.2-83.6)	79.2 (76.9-81.5)	74.9 (72.6-77.4)	73.4 (70.7-76.1)	68.6 (66.0-71.3)	64.1 (61.3-67.6)	<.001
400-m walk time[e]	696	59.4 (56.0-63.2)	64.2 (60.1-67.7)	61.0 (56.4-64.9)	62.3 (57.9-66.0)	58.7 (55.3-62.3)	50.2 (45.3-54.8)	0.02[†]
Mobility deficit remission	172	48.4 (41.0-55.5)	52.0 (44.6-58.7)	49.8 (42.4-57.4)	47.4 (39.2-54.1)	42.5 (35.0-50.2)	40.7 (32.4-49.0)	0.11
WOMAC								
Knee pain[f]	334	80.1 (75.2-84.3)	76.7 (72.2-81.0)	76.7 (72.3-80.9)	75.6 (71.2-80.2)	70.0 (64.9-74.8)	69.4 (64.4-75.0)	0.01
Hip pain[f]	251	76.7 (71.8-81.6)	75.2 (70.1-80.2)	77.3 (72.4-82.4)	74.4 (68.6-79.6)	67.6 (62.4-73.6)	64.7 (58.1-71.7)	<.001
Knee function[g]	337	82.3 (78.1-86.2)	81.5 (77.4-85.3)	77.4 (73.1-81.7)	77.2 (73.1-81.6)	71.3 (66.8-76.2)	71.6 (66.6-77.3)	0.04
Hip function[g]	251	80.4 (75.7-84.8)	80.8 (75.6-85.3)	78.4 (72.9-83.5)	74.7 (69.2-80.0)	70.5 (64.7-76.0)	65.5 (58.7-72.5)	0.001

Abbreviations: RYGB, Roux-en-Y gastric bypass; SF-36, Medical Outcomes Study Short-Form 36 Health Survey; SG, sleeve gastrectomy; WOMAC, Western Ontario and McMaster Universities Osteoarthritis Index.

[a] Improvements in bodily pain and physical function were evaluated among the entire cohort; 400-m walk time improvement was evaluated among participants who completed the walk preoperative; improvement in knee or hip pain and function were evaluated among those with severe pain or disability preoperative; remission of mobility deficit was evaluated among those with a mobility deficit preoperative.

[b] All models were adjusted for site and age.

[c] P-values from linear trend test are reported if P-value from quadratic trend ≥.05. † indicates quadratic trend test p-value is reported.

[d] Norm-based score improvement (increase) of at least 5 points.

[e] Time indicates improvement (decrease) of at least 24 seconds.

[f] Score improvement (decrease) of at least 9.7 pain points. Twelve of 459 participants with preoperative symptoms of osteoarthritis of the knee and 17 of 347 participants with preoperative symptoms of osteoarthritis of the hip were excluded from analysis because their preoperative pain score was less than 9.7 points.

[g] Score improvement (decrease) of at least 9.3 function points. Five of 459 participants with preoperative symptoms of osteoarthritis of the knee and 7 of 347 participants with preoperative symptoms of osteoarthritis of the hip were excluded from analysis because their preoperative function score was less than 9.3 points.

© 2022 King WC et al. *JAMA Network Open.*

eTable 7. Descriptive Statistics of Work Productivity Measures by Time in Relation to RYGB or SG (N=693[a])

	No./total No. (%)[b]						
	**Preoperative**	**Year 1**	**Year 2**	**Year 3**	**Year 4**	**Year 5**	**Year 7**
**Work Productivity**							
Absenteeism (missed work due to health)	103/644 (16.0%)	49/534 (9.2%)	50/486 (10.3%)	61/490 (12.4%)	57/481 (11.9%)	58/505 (11.5%)	52/369 (14.1%)
Percentage of work missed due to health[c]	n=619	n=513	n=463	n=470	n=453	n=483	n=355
0	516 (83.4%)	465 (90.6%)	414 (89.4%)	409 (87.0%)	396 (87.4%)	425 (88.0%)	303 (85.4%)
0.1-10.0	36 (5.8%)	13 (2.5%)	16 (3.5%)	22 (4.7%)	15 (3.3%)	13 (2.7%)	15 (4.2%)
10.1-20.0	28 (4.5%)	12 (2.3%)	8 (1.7%)	19 (4.0%)	13 (2.9%)	12 (2.5%)	13 (3.7%)
20.1-30.0	16 (2.6%)	5 (1.0%)	9 (1.9%)	6 (1.3%)	5 (1.1%)	9 (1.9%)	9 (2.5%)
30.1-40.0	6 (1.0%)	6 (1.2%)	3 (0.6%)	6 (1.3%)	8 (1.8%)	5 (1.0%)	2 (0.6%)
40.1-50.0	5 (0.8%)	5 (1.0%)	2 (0.4%)	1 (0.2%)	8 (1.8%)	2 (0.4%)	1 (0.3%)
>50	12 (1.9%)	7 (1.4%)	11 (2.4%)	7 (1.5%)	8 (1.8%)	17 (3.5%)	12 (3.4%)
Presenteeism (impairment while working due to health)	389/615 (63.3%)	139/512 (27.1%)	143/461 (31.0%)	170/462 (36.8%)	155/452 (34.3%)	185/474 (39.0%)	151/348 (43.4%)
Percentage of time working impaired due to health[d]	n=615	n=512	n=461	n=462	n=452	n=474	n=348
0	226 (36.7%)	373 (72.9%)	318 (69.0%)	292 (63.2%)	297 (65.7%)	289 (61.0%)	197 (56.6%)
0.1-10.0	110 (17.9%)	55 (10.7%)	54 (11.7%)	58 (12.6%)	44 (9.7%)	57 (12.0%)	39 (11.2%)
10.1-20.0	84 (13.7%)	29 (5.7%)	32 (6.9%)	48 (10.4%)	36 (8.0%)	48 (10.1%)	38 (10.9%)
20.1-30.0	74 (12.0%)	27 (5.3%)	18 (3.9%)	24 (5.2%)	29 (6.4%)	31 (6.5%)	24 (6.9%)
30.1-40.0	31 (5.0%)	9 (1.8%)	11 (2.4%)	16 (3.5%)	14 (3.1%)	15 (3.2%)	15 (4.3%)
40.1-50.0	31 (5.0%)	6 (1.2%)	5 (1.1%)	9 (1.9%)	11 (2.4%)	9 (1.9%)	13 (3.7%)
>50	59 (9.6%)	13 (2.5%)	23 (5.0%)	15 (3.2%)	21 (4.6%)	25 (5.3%)	22 (6.3%)

[a]Among 1491 participants in this report, 796 participants were excluded from work productivity measures because they were not employed at all non-missing assessments and 2 were excluded because they did not answer relevant questions on the Work Productivity and Activity Impairment questionnaire.
[b]Unless otherwise indicated.
[c]Participants recorded the number of hours worked, missed due to health, and missed due to other reasons. Absenteeism is defined as any work missed due to health. The percentage of work missed due to health was calculated as hours missed from work "because of your health problems" divided by the sum of hours missed for any reason plus hours worked.
[d]Participants indicated how much their health problems affected their productivity via a rating scale (0 to 10; 0 = "health problems had no effect on my work" to 10 = "health problems completely prevented me from working"). A rating greater than 0 indicates presenteeism (i.e., health problems affecting work productivity). The response times 10 is assumed to represent a percentage reduction in productive work due to health problems.

© 2022 King WC et al. *JAMA Network Open.*

eTable 8. Descriptive Statistics of Pain Measures by Time in Relation to SG (n=51[a])

	No./total No. (%)[a]						
	**Preoperative**	**Year 1**	**Year 2**	**Year 3**	**Year 4**	**Year 5**	**Year 7**
**SF-36 Score[b]**							
Bodily Pain	n=48	n=44	n=44	n=39	n=32	n=41	n=31
Median (25th- 75th percentile)	35.4 (35.4-44.3)	46.4 (35.4-60.4)	44.3 (35.4-53.6)	44.3 (35.4-53.6)	39.8 (35.4-53.6)	40.0 (35.4-53.6)	40.0 (35.4-49.4)
**Back or Leg Pain**							
Medication for back pain, past week	15/45 (33.3)	12/44 (27.3)	10/40 (25.0)	10/37 (27.0)	8/32 (25.0)	13/43 (30.2)	10/30 (33.3)
Medication for leg pain, past week	18/46 (39.1)	12/44 (27.3)	14/42 (33.3)	12/37 (32.4)	13/30 (43.3)	15/43 (34.9)	7/31 (22.6)
Back or leg pain interfered with work (outside the home or house work), past 4 weeks, n (%)	n=45	n=43	n=42	n=38	n=31	n=40	n=31
Not at all	23 (51.1)	28 (65.1)	29 (69.0)	26 (68.4)	17 (54.8)	22 (55.0)	23 (74.2)
A little bit	3 (6.7)	5 (11.6)	5 (11.9)	3 (7.9)	4 (12.9)	6 (15.0)	1 (3.2)
Moderately	7 (15.6)	3 (7.0)	3 (7.1)	3 (7.9)	6 (19.4)	6 (15.0)	2 (6.5)
Quite a bit	6 (13.3)	7 (16.3)	5 (11.9)	2 (5.3)	3 (9.7)	5 (12.5)	4 (12.9)
Extremely	6 (13.3)	0 (0.0)	0 (0.0)	4 (10.5)	1 (3.2)	1 (2.5)	1 (3.2)
Could not go to work or school due to back or leg pain, past 4 weeks	5/46 (10.9)	3/43 (7.0)	1/42 (2.4)	2/37 (5.4)	1/31 (3.2)	3/41 (7.3)	3/31 (9.7)
Feelings regarding back or leg pain, n (%)	n=46	n=43	n=42	n=38	n=31	n=41	n=32
Very dissatisfied	14 (30.4)	6 (14.0)	6 (14.3)	6 (15.8)	6 (19.4)	6 (14.6)	2 (6.3)
Dissatisfied	7 (15.2)	3 (7.0)	5 (11.9)	2 (5.3)	4 (12.9)	6 (14.6)	2 (6.3)
Somewhat dissatisfied	1 (2.2)	5 (11.6)	3 (7.1)	5 (13.2)	3 (9.7)	4 (9.8)	3 (9.4)
Neither satisfied or dissatisfied	1 (2.2)	3 (7.0)	3 (7.1)	2 (5.3)	3 (9.7)	2 (4.9)	2 (6.3)
Somewhat satisfied	1 (2.2)	1 (2.3)	1 (2.4)	0 (0.0)	0 (0.0)	1 (2.4)	1 (3.1)
Satisfied	0 (0.0)	2 (4.7)	0 (0.0)	1 (2.6)	1 (3.2)	1 (2.4)	1 (3.1)
Very satisfied	22 (47.8)	23 (53.5)	24 (57.1)	22 (57.9)	14 (45.2)	21 (51.2)	21 (65.6)
Back pain during 400-m walk[c]	14/37 (37.8)	7/40 (17.5)	5/33 (15.2)	6/33 (18.2)	5/29 (17.2)	2/24 (8.3)	4/19 (21.1)
Leg pain during 400-m walk[c]	23/37 (62.2)	8/40 (20.0)	6/33 (18.2)	7/33 (21.2)	8/29 (27.6)	9/24 (37.5)	7/19 (36.8)
**WOMAC Scores[d]**							
Knee pain[e]	n=21	n=15	n=17	n=16	n=14	n=18	n=10
Median (25th- 75th percentile)	50.0 (40.0-65.0)	20.0 (0.0-60.0)	25.0 (10.0-50.0)	30.0 (17.5-45.0)	22.5 (10.0-35.0)	30.0 (25.0-50.0)	30.0 (10.0-45.0)
Hip pain[f]	n=14	n=9	n=9	n=10	n=8	n=12	n=4
Median (25th- 75th percentile)	60.0 (45.0-65.0)	30.0 (25.0-50.0)	55.0 (25.0-60.0)	47.5 (40.0-60.0)	37.5 (17.5-57.5)	45.0 (15.0-55.0)	27.5 (2.5-57.5)

Abbreviations: SF-36, 36-item short form health survey; SG, sleeve gastrectomy; WOMAC, Western Ontario and McMaster Universities osteoarthritis index.
[a] Unless otherwise indicated.
[b] Norm-based methods were used to transform scores (mean [SD], 50 [10]) in the general US population. Higher scores indicate less pain.
[c] N =37; excludes participants who did not start the 400-m walk preoperative.
[d] Lower scores indicate less pain on a 0- to 100-point scale.
[e] N=21; excludes participants who did not have preoperative symptoms of osteoarthritis of the knee.
[f] N=14; excludes participants who did not have preoperative symptoms of osteoarthritis of the hip.

© 2022 King WC et al. *JAMA Network Open.*

eTable 9. Descriptive Statistics of Physical Function Measures by Time in Relation to SG (n=51)

	No./total No. (%)[a]						
	**Preoperative**	**Year 1**	**Year 2**	**Year 3**	**Year 4**	**Year 5**	**Year 7**
**SF-36 Score**[b]							
Physical function	n=48	n=44	n=44	n=38	n=32	n=41	n=30
Median (25th- 75th percentile)	30.6 (21.1-39.3)	52.4 (42.6-54.6)	50.2 (39.5-54.6)	49.1 (32.7-54.6)	46.9 (30.6-53.5)	48.0 (37.1-54.6)	43.7 (26.2-54.6)
**Self-Reported Walking**							
Severe walking limitation	5/41 (12.2)	5/39 (12.8)	3/35 (8.6)	5/35 (14.3)	7/29 (24.1)	9/40 (22.5)	5/29 (17.2)
Mobility aid use	14/46 (30.4)	12/40 (30.0)	11/39 (28.2)	10/36 (27.8)	10/30 (33.3)	13/38 (34.2)	10/32 (31.3)
Health limits ability to:							
Walk 1 block	27/48 (56.3)	5/44 (11.4)	9/44 (20.5)	8/38 (21.1)	7/32 (21.9)	8/41 (19.5)	9/29 (31.0)
Walk several blocks	41/48 (85.4)	10/44 (22.7)	11/44 (25.0)	13/37 (35.1)	9/31 (29.0)	14/41 (34.1)	11/29 (37.9)
Walk >1 mile	44/48 (91.7)	21/44 (47.7)	22/44 (50.0)	19/37 (51.4)	17/32 (53.1)	20/41 (48.8)	17/30 (56.7)
**400-m Walk Test**							
Completed	31/48 (64.6)	40/44 (90.9)	33/39 (84.6)	32/36 (88.9)	29/35 (82.9)	26/35 (74.3)	16/27 (59.3)
Mobility deficit	26/48 (54.2)	13/44 (29.5)	10/39 (25.6)	6/35 (17.1)	12/34 (35.3)	10/33 (30.3)	8/23 (34.8)
Time to complete, seconds[c]	n=31	n=25	n=23	n=23	n=21	n=20	n=15
Median	381.9	364.0	345.8	356.0	343.9	350.3	351.0
(25th- 75th percentile)	(336.7-435.3)	(325.0-392.8)	(315.7-368.4)	(327.6-381.3)	(318.8-394.4)	(327.5-382.3)	(326.3-379.8)
**Fitness Proxy**							
Resting heart rate, bpm[d]	n=33	n=35	n=33	n=36	n=31	n=30	n=24
Median (25th- 75th percentile)	82 (74-86)	70 (58-82)	70 (64-79)	72 (65-80)	74 (67-82)	73 (66-81)	72 (63.5-80)
**WOMAC Scores**[e]							
Knee physical function[f]	n=21	n=15	n=17	n=15	n=13	n=18	n=10
Median (25th- 75th percentile)	48.5 (35.3-61.7)	11.8 (0.0-54.4)	27.9 (4.4-41.2)	25.0 (16.2-51.6)	16.2 (9.3-35.3)	26.5 (13.2-45.6)	25.0 (7.4-42.6)
Hip physical function[g]	n=14	n=9	n=9	n=10	n=8	n=12	n=4
Median (25th- 75th percentile)	58.1 (32.3-63.2)	38.2 (16.2-48.5)	39.7 (16.2-64.7)	45.3 (26.5-58.8)	30.1 (12.5-53.7)	41.2 (14.7-54.4)	27.9 (2.9-63.2)

Abbreviations: SF-36, 36-item short form health survey; SG, sleeve gastrectomy; WOMAC, Western Ontario and McMaster Universities osteoarthritis index; bpm, beats per minute.
[a] Unless otherwise indicated.
[b] Norm-based methods were used to transform scores (mean [SD], 50 [10]) in the general US population. Higher scores indicate better function.
[c] N=31; excludes 17 of 48 participants who did not complete the 400-m walk at preoperative due to ineligibility, refusal, or meeting stopping criteria.
[d] Data set to missing at assessments at which participants were taking β-blockers.
[e] Lower scores indicate better function on a 0- to 100-point scale.
[f] N=21; excludes participants who did not have preoperative symptoms of osteoarthritis of the knee.
[g] N=14; excludes participants who did not have preoperative symptoms of osteoarthritis of the hip.

© 2022 King WC et al. *JAMA Network Open.*

eTable 10. Observed Percentage of Patients With Clinically Important Improvements in Pain and Physical Function [a] by Year Since SG

	No./total No. (%)					
	**Year 1**	**Year 2**	**Year 3**	**Year 4**	**Year 5**	**Year 7**
**SF-36 Scores**[b]						
Bodily Pain	25/42 (59.5)	24/42 (57.1)	22/37 (59.5)	17/31 (54.8)	17/39 (43.6)	15/31 (48.4)
Physical function	35/42 (83.3)	34/42 (81.0)	27/36 (75.0)	21/31 (67.7)	31/39 (79.5)	19/30 (63.3)
400-m walk time[c]	14/25 (56.0)	13/23 (56.5)	15/23 (65.2)	10/21 (47.6)	10/20 (50.0)	6/15 (40.0)
Mobility deficit remission	1/12 (8.3)	3/12 (25.0)	4/12 (33.3)	0/9 (0.0)	1/13 (7.7)	2/8 (25.0)
**WOMAC Scores**						
Knee pain[d]	10/14 (71.4)	12/16 (75.0)	12/15 (80.0)	11/13 (84.6)	12/17 (70.6)	6/9 (66.7)
Hip pain[d]	7/8 (87.5)	5/8 (62.5)	7/9 (77.8)	6/7 (85.7)	8/11 (72.7)	3/4 (75.0)
Knee physical function[e]	11/15 (73.3)	11/17 (64.7)	12/15 (80.0)	10/13 (76.9)	12/18 (66.7)	6/10 (60.0)
Hip physical function[e]	7/9 (77.8)	4/9 (44.4)	6/10 (60.0)	5/8 (62.5)	8/12 (66.7)	3/4 (75.0)

Abbreviations: SF-36, Medical Outcomes Study Short-Form 36 Health Survey; SG, sleeve gastrectomy; WOMAC, Western Ontario and McMaster Universities Osteoarthritis Index.
[a] Improvements in bodily pain and physical function were evaluated among the entire cohort; 400-m walk time improvement was evaluated among participants who completed the walk preoperative; improvement in knee or hip pain and function were evaluated among those with severe pain or disability preoperative; remission of mobility deficit was evaluated among those with a mobility deficit preoperative.
[b] Norm-based score improvement (increase) of at least 5 points.
[c] Improvement (decrease) of at least 24 seconds.
[d] Score improvement (decrease) of at least 9.7 pain points. One of 21 participants with preoperative symptoms of osteoarthritis of the knee and 2 of 14 participants with preoperative symptoms of osteoarthritis of the hip were excluded from analysis because their preoperative score was less than 9.7 points.
[e] Score improvement (decrease) of at least 9.3 function points.

© 2022 King WC et al. *JAMA Network Open.*

eTable 11. Descriptive Statistics of Work Productivity Measures by Time in Relation to SG (n=23[a]).

	No./total No. (%)[b]						
	**Preoperative**	**Year 1**	**Year 2**	**Year 3**	**Year 4**	**Year 5**	**Year 7**
**Work Productivity**							
Absenteeism (missed work due to health)	4/23 (17.4%)	1/17 (5.9%)	1/17 (5.9%)	1/16 (6.3%)	1/13 (7.7%)	0/16 (0.0%)	2/13 (15.4%)
Percentage of work missed due to health[c]	n=22	n=17	n=16	n=16	n=13	n=16	n=13
0	18 (81.8%)	16 (94.1%)	15 (93.8%)	15 (93.8%)	12 (92.3%)	16 (100.0%)	11 (84.6%)
0.1-10.0	1 (4.5%)	0 (0.0%)	0 (0.0%)	0 (0.0%)	0 (0.0%)	0 (0.0%)	1 (7.7%)
10.1-20.0	2 (9.1%)	0 (0.0%)	1 (6.3%)	0 (0.0%)	0 (0.0%)	0 (0.0%)	0 (0.0%)
20.1-30.0	1 (4.5%)	0 (0.0%)	0 (0.0%)	0 (0.0%)	1 (7.7%)	0 (0.0%)	1 (7.7%)
30.1-40.0	0 (0.0%)	0 (0.0%)	0 (0.0%)	0 (0.0%)	0 (0.0%)	0 (0.0%)	0 (0.0%)
40.1-50.0	0 (0.0%)	1 (5.9%)	0 (0.0%)	0 (0.0%)	0 (0.0%)	0 (0.0%)	0 (0.0%)
>50	0 (0.0%)	0 (0.0%)	0 (0.0%)	1 (6.3%)	0 (0.0%)	0 (0.0%)	0 (0.0%)
Presenteeism (impairment while working due to health)	15/22 (68.2%)	6/17 (35.3%)	7/18 (38.9%)	6/15 (40.0%)	6/14 (42.9%)	7/15 (46.7%)	8/13 (61.5%)
Percentage of time working impaired due to health[d]	n=22	n=17	n=18	n=15	n=14	n=15	n=13
0	7 (31.8%)	11 (64.7%)	11 (61.1%)	9 (60.0%)	8 (57.1%)	8 (53.3%)	5 (38.5%)
0.1-10.0	3 (13.6%)	5 (29.4%)	5 (27.8%)	4 (26.7%)	4 (28.6%)	3 (20.0%)	2 (15.4%)
10.1-20.0	4 (18.2%)	0 (0.0%)	2 (11.1%)	2 (13.3%)	0 (0.0%)	3 (20.0%)	0 (0.0%)
20.1-30.0	2 (9.1%)	1 (5.9%)	0 (0.0%)	0 (0.0%)	0 (0.0%)	0 (0.0%)	3 (23.1%)
30.1-40.0	2 (9.1%)	0 (0.0%)	0 (0.0%)	0 (0.0%)	1 (7.1%)	0 (0.0%)	1 (7.7%)
40.1-50.0	2 (9.1%)	0 (0.0%)	0 (0.0%)	0 (0.0%)	0 (0.0%)	1 (6.7%)	2 (15.4%)
>50	2 (9.1%)	0 (0.0%)	0 (0.0%)	0 (0.0%)	1 (7.1%)	0 (0.0%)	0 (0.0%)

[a]Among 51 participants in this report, 28 were excluded from work productivity measures because they were not employed at all non-missing assessments.
[b]Unless otherwise indicated.
[c]Participants recorded the number of hours worked, missed due to health, and missed due to other reasons. Absenteeism is defined as any work missed due to health. The percentage of work missed due to health was calculated as hours missed from work "because of your health problems" divided by the sum of hours missed for any reason plus hours worked.
[d]Participants indicated how much their health problems affected their productivity via a rating scale (0 to 10; 0 = "health problems had no effect on my work" to 10 = "health problems completely prevented me from working"). A rating greater than 0 indicates presenteeism (i.e., health problems affecting work productivity). The response times 10 is assumed to represent a percentage reduction in productive work due to health problems.

© 2022 King WC et al. *JAMA Network Open.*
